# Susceptibility of oral bacteria to antibacterial photodynamic therapy

**DOI:** 10.1080/20002297.2019.1644111

**Published:** 2019-07-19

**Authors:** Si-Mook Kang, Hoi-In Jung, Baek-Il Kim

**Affiliations:** Department of Preventive Dentistry & Public Oral Health, BK 21 PLUS Project, Yonsei University College of Dentistry, Seoul, Republic of Korea

**Keywords:** Antibacterial photodynamic therapy, oral bacteria, prevention, photosensitizer, visible violet-blue light

## Abstract

Effective methods for managing the oral microbiome are necessary to ensure not only the oral but also the systemic health of a human body. The purpose of this study was to determine the sensitivity of four photosensitizers (PSs) to blue light in six representative oral bacterial species that cause intraoral diseases. The following six strains were investigated: *Actinomyces israelii*,* Enterococcus faecium*, *Fusobacterium nucleatum*, *Lactobacillus gasseri*, *Streptococcus mutans,* *Veillonella parvula*. PS stock solutions (1 mg/ml) were prepared by dissolving curcumin and protoporphyrin-IX in dimethyl sulfoxide, and resazurin and riboflavin in distilled water. The inoculation of 20 ml of a bacterial suspension cultured for 24 hours was mixed with 1,980 ml of each test solution, and then a light source was placed in front of the mixture. The irradiation wavelength was 405 nm and its applied energy was 25.3 J. The independent-samples *t*-test and one-way analysis of variance within groups were performed to compare the antibacterial effects in the four PSs. The antibacterial susceptibility when using different PSs and visible blue-light irradiation differed between the bacterial strains. Antibacterial photodynamic therapy that includes light exposure and PSs can be used to control the oral bacteria strains related to oral disease.

The oral cavity is one of most complex organs, consisting of various structures including teeth, tongue, and cheek tissue. More than 600 species or phylotypes reside in the oral cavity and many systemic diseases are related to the composition of the oral microbiota [1,2], which makes effective methods for managing the oral microbiome necessary to ensure the systemic health of a human body. Brushing is the most widely used method to control the bacteria in the oral cavity, but some areas such as pits and fissures of molars are difficult to clean [3]. Attempts to overcome this limitation have included modifying the shape of toothbrush bristles and using oral care products such as oral rinses and dental floss. In addition, many germs are present on the tongue, but the area near the throat is very difficult to clean due to the gag reflex [4]. The presence of dental structures with various shapes and sizes, such as implants, resins, brackets, and dentures, make the oral cavity complex. Microleakages occur at the interface between the teeth and these materials, which may cause diseases such as secondary caries and implant failure.

In the early 1900s, von Tappeiner and his colleague Oscar Raab discovered that a substance called Acridine could kill paramecia upon light irradiation, and in combination with oxygen they introduced the term ‘photodynamic action’ for this phenomenon [5]. Photodynamic therapy (PDT) using a photosensitizer (PS) and light is easy to implement and apply in many fields. In particular, antibacterial PDT (aPDT) has many advantages as a new alternative to antibiotics because it does not induce the tolerance in bacteria that has become a major problem in recent years [6–8]. PDT has also been shown to be highly effective in the field of dentistry [9]. The antibacterial effects of PDT on bacteria associated with peri-implantitis, periodontitis, and caries pathogens have also been reported [10–12]. However, most of these studies used excitation light with a wavelength longer than 500 nm. A longer wavelength will increase the penetration depth but decrease the light energy, hence reducing the clinical antimicrobial effects. In addition, few studies have investigated the sensitivity of bacteria to different types of PS. The purpose of the present study was to determine the sensitivity of four PSs to violet-blue light in six representative oral bacteria species that cause intraoral diseases.

## Materials and methods

### Bacterial strains and culture media

The following six strains that are known to be associated with oral diseases were investigated: *Streptococcus mutans* ATCC 25,175 and *Lactobacillus gasseri* ATCC 33,323 associated with dental caries; *Enterococcus faecium* ATCC 19,434, *Fusobacterium nucleatum* ATCC 25,586, and *Veillonella parvula* ATCC 10,790 associated with periodontal disease; and *Actinomyces israelii* ATCC 10,049 associated with actinomycosis. All six strains were purchased from the Korean Collection for Type Cultures (Biological Resource Center, Korea). *A. israelii, E. faecium*, and *F. nucleatum* were grown in thioglycollate broth; *L. gasseri* was grown in MRS (de Man, Rogosa, and Sharpe) broth; and *S. mutans* and *V. parvula* were growth in BHI (brain heart infusion) broth. The composition of each broth is listed in Table 1. All liquid broths were incubated anaerobically under 80% N_2_, 10% H_2_, and 10% CO_2_ at 37°C.

**Table 1.** Compositions of the bacterial media used in this study.

Component	BHI broth	MRS broth	Thioglycollate broth
Beef extract	-	10.0 g/l	-
Casitone	-	-	15.0 g/l
K_2_HPO_4_	-	2.0 g/l	-
L-cysteine hydrochloride	-	-	0.25 g/l
MgSO_4_ · 7H_2_O	-	0.2 g/l	-
MnSO_4_ · 4H_2_O	-	0.2 g/l	-
Peptone	-	10.0 g/l	-
Sodium acetate	-	5.0 g/l	-
Sodium chloride	5.0 g/l	-	2.5 g/l
Sodium thioglycolate	-	-	0.5 g/l
Triammonium citrate	-	2.0 g/l	-
Tween 80	-	1.0 ml/l	-
Yeast extract	-	5.0 g/l	5.0 g/l
BHI	17.5 g/l	-	-
Disodium phosphate	2.5 g/l	-	-
Enzymatic digest of gelatin	10.0 g/l	-	-
Glucose (dextrose)	2.0 g/l	20.0 g/l	6.0 g/l
Lactic acid	4.5 g/l	-	-
0.05% Hemin solution	-	-	10 ml/l
0.5% Vitamin K_1_ solution	-	-	0.2 ml/l
pH	7.4 ± 0.2	6.4	7.1 ± 0.2

### Photodynamic treatment

For the photodynamic treatment, stock solutions of four PSs were prepared and stored at a concentration of 1 mg/ml. Powder forms of curcumin (MW:368.38) and protoporphyrin IX (MW:606.62) were dissolved in dimethyl sulfoxide (since they have low solubility in water), and those of resazurin (MW:251.17) and riboflavin (MW:376.36) were dissolved in distilled water. To evaluate the antimicrobial activity induced by photodynamic reactions, a stock solution was diluted tenfold in a liquid medium to which each strain could be cultured to prepare a solution having a concentration of 100 μg/ml. Serial dilution was then used to prepare test solutions with concentrations of 10 μg/ml, 1 μg/ml, and 0.1 μg/ml. The antimicrobial activity against light irradiation was evaluated by preparing 1,980 μl of test solutions in two 24-well plates, and 20 μl of 24 h cultured medium. One plate was irradiated for 5 min and the other plate was placed in a dark condition. The light irradiation for inducing the photodynamic reaction was provided by a QLF-D (quantitative light-induced fluorescence – digital, Inspektor Research Systems, Amsterdam, Netherlands) device that included a 405-nm LED, and the total light energy was 25.3 J (84.5 mW/cm^2^ × 300 s).

After treatment, a 200-μl aliquot of each solution was transferred to a 96-well plate and incubated at 37°C in an anaerobic incubator for 24 h. The absorbance of the incubated solution was measured in a 96-well microplate reader (Model 680, Bio-Rad, Hercules, CA) at 655 nm. Each experiment studying antibacterial activity was repeated three times.

### Statistical analyses

Statistical analysis was performed using one-way analysis of variance and the Tukey test to detect differences in the bactericidal activity of PSs at different concentrations against *S. mutans*. In addition, Student’s *t*-test was used for comparisons between with and without light irradiation. PASW Statistics (version 23.0, SPSS, IBM Corporation, Armonk, NY) was used for all data analyses, with a significance cutoff of *p* < 0.05. 

## Results

**Figure 1.**
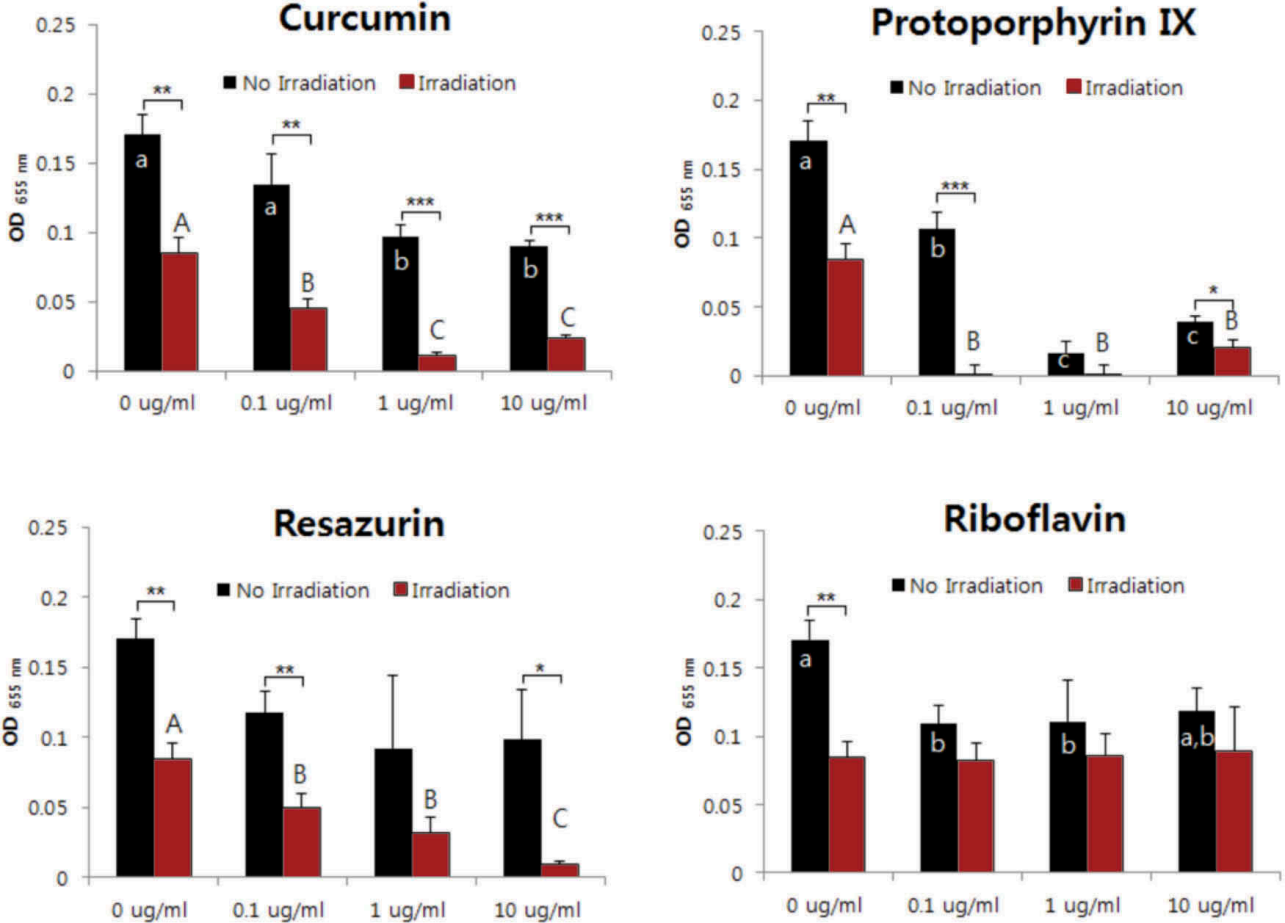
Photodynamic inactivation of *A. israelii* by four PSs. *p < 0.05, **p < 0.01, ***p < 0.001. Different letters indicate significant differences between groups (*p* < 0.05). Data are mean and SD values.

**Figure 2.**
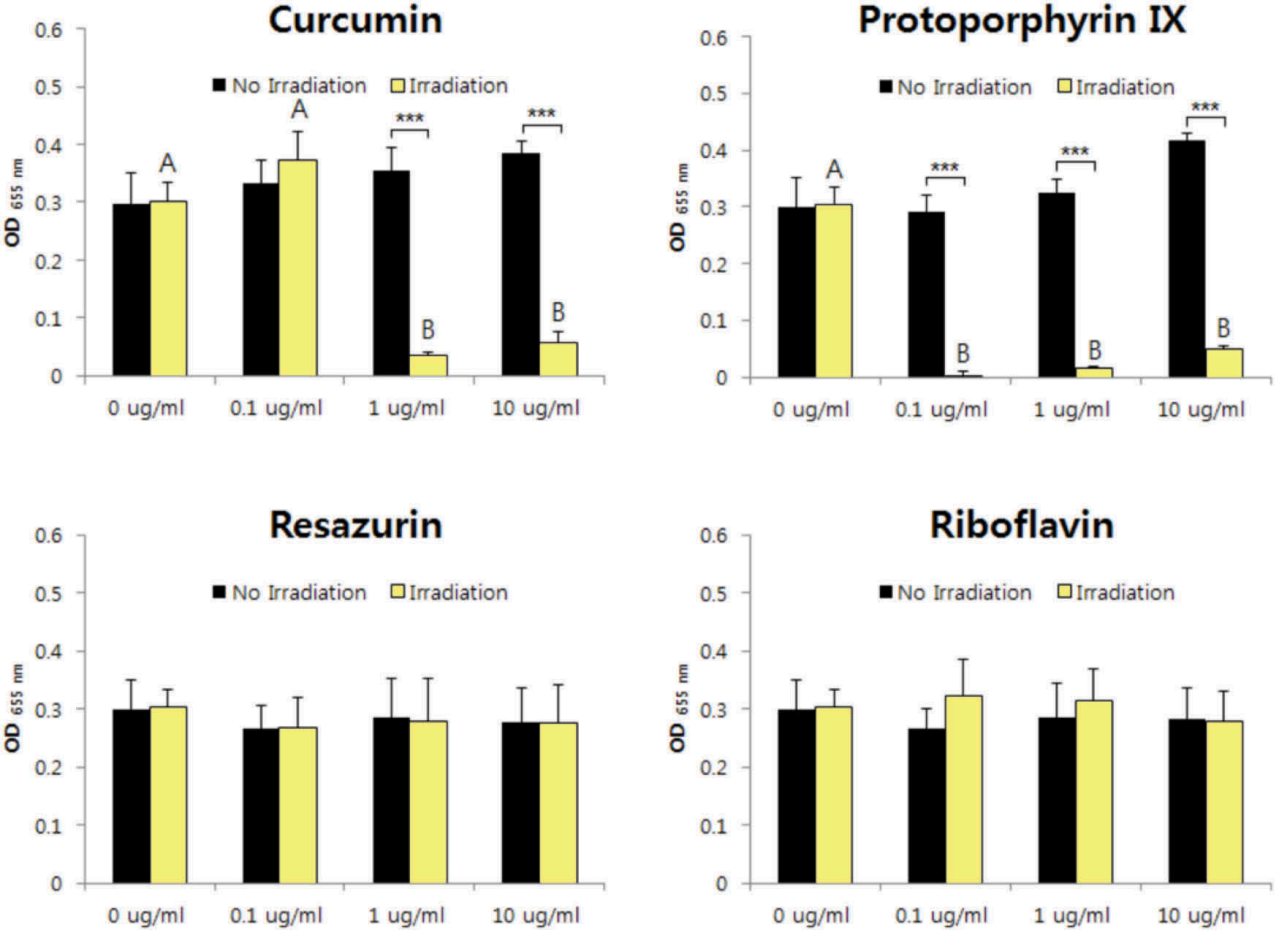
Photodynamic inactivation of *E. faecium* by four PSs. ***p < 0.001. Different letters indicate significant differences between groups (*p* < 0.05). Data are mean and SD values.

**Figure 3.**
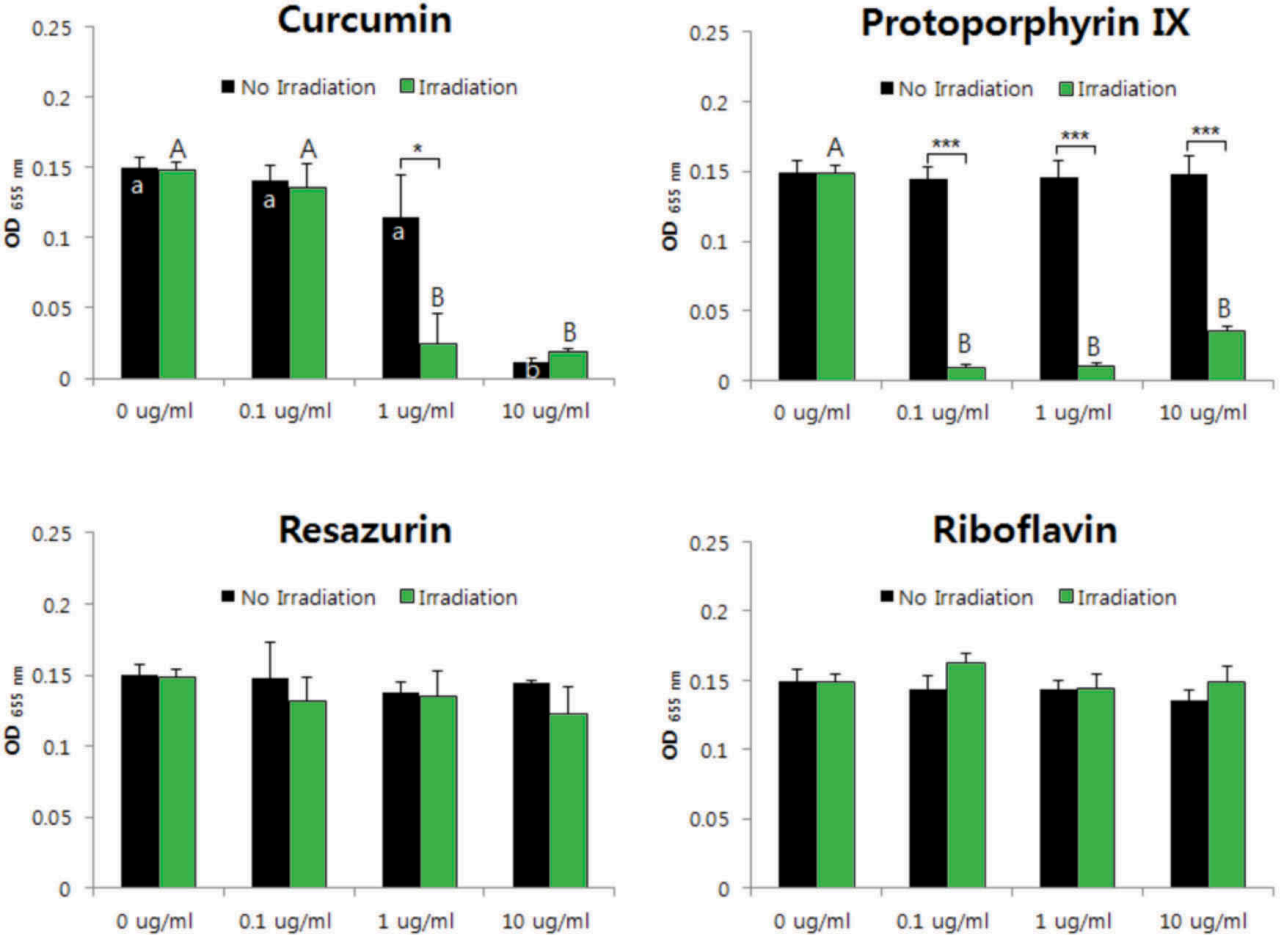
Photodynamic inactivation of *F. nucleatum* by four PSs. *p < 0.05, ***p < 0.001. Different letters indicate significant differences between groups (*p* < 0.05). Data are mean and SD values.

**Figure 4.**
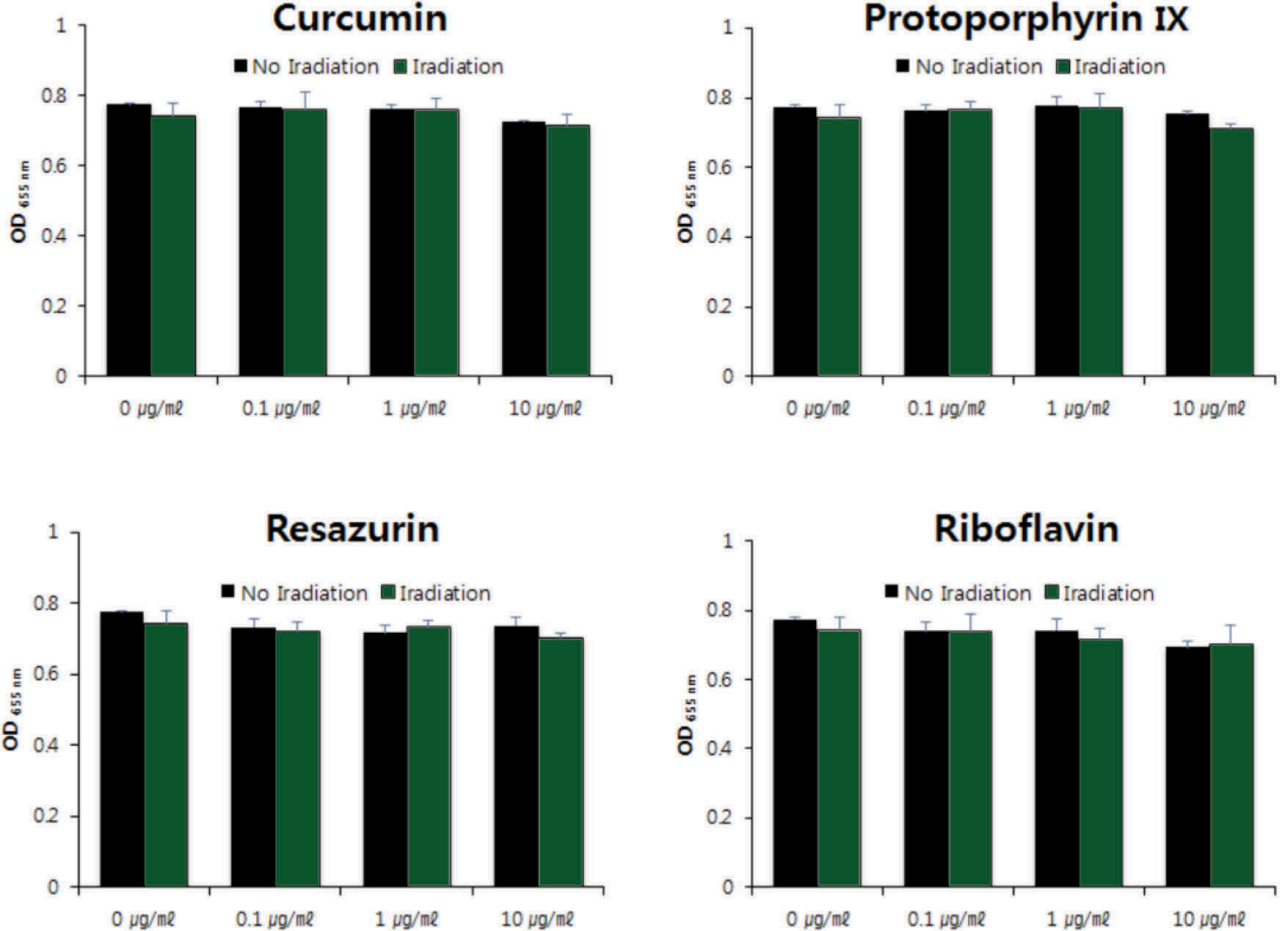
Photodynamic inactivation of *S. mutans* by four PSs. ***p < 0.001. Different letters indicate significant differences between groups (*p* < 0.05). Data are mean and SD values.

**Figure 5.**
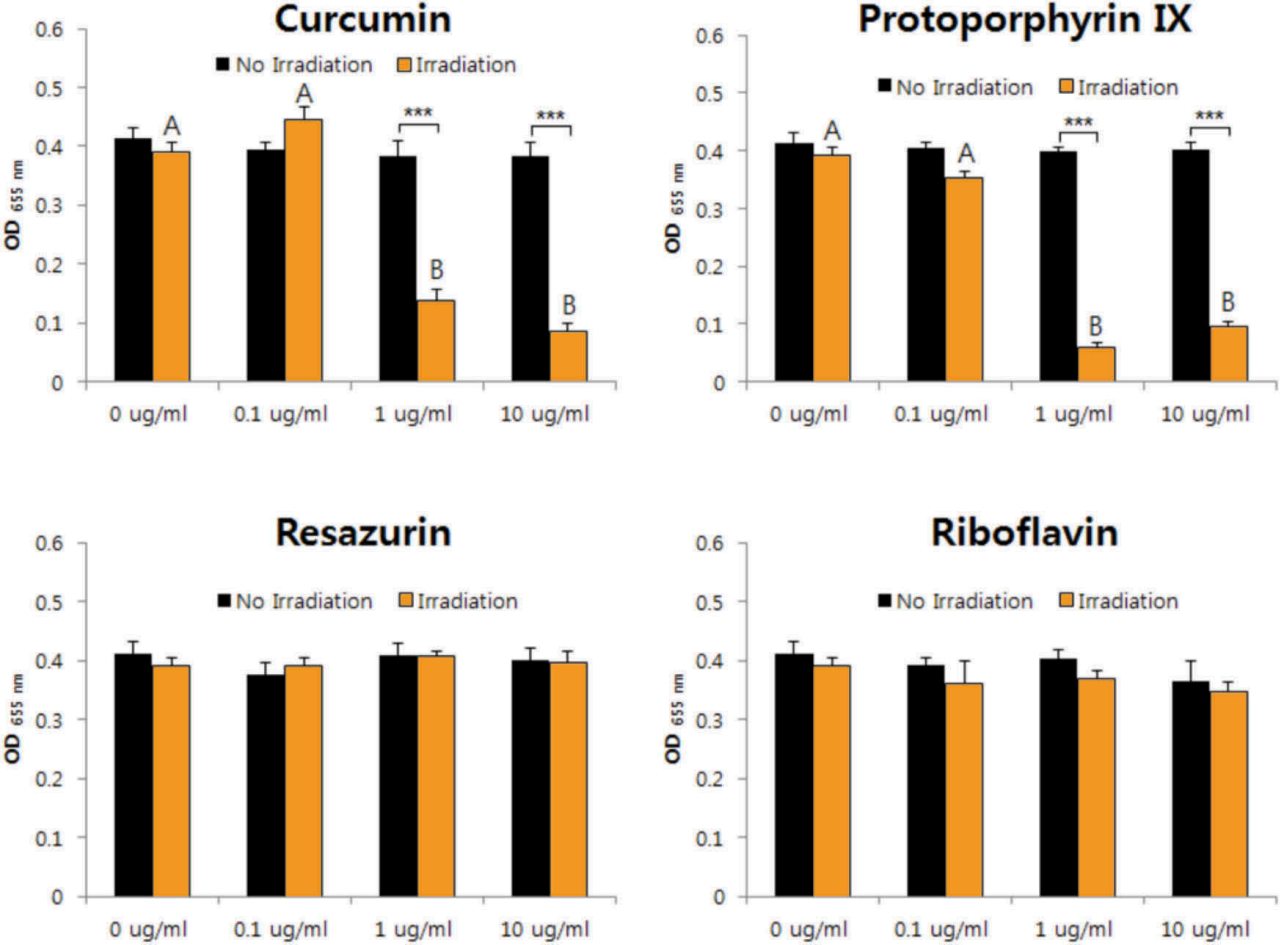
Photodynamic inactivation of *L. gasseri* by four PSs. Data are mean and SD values.

**Figure 6.**
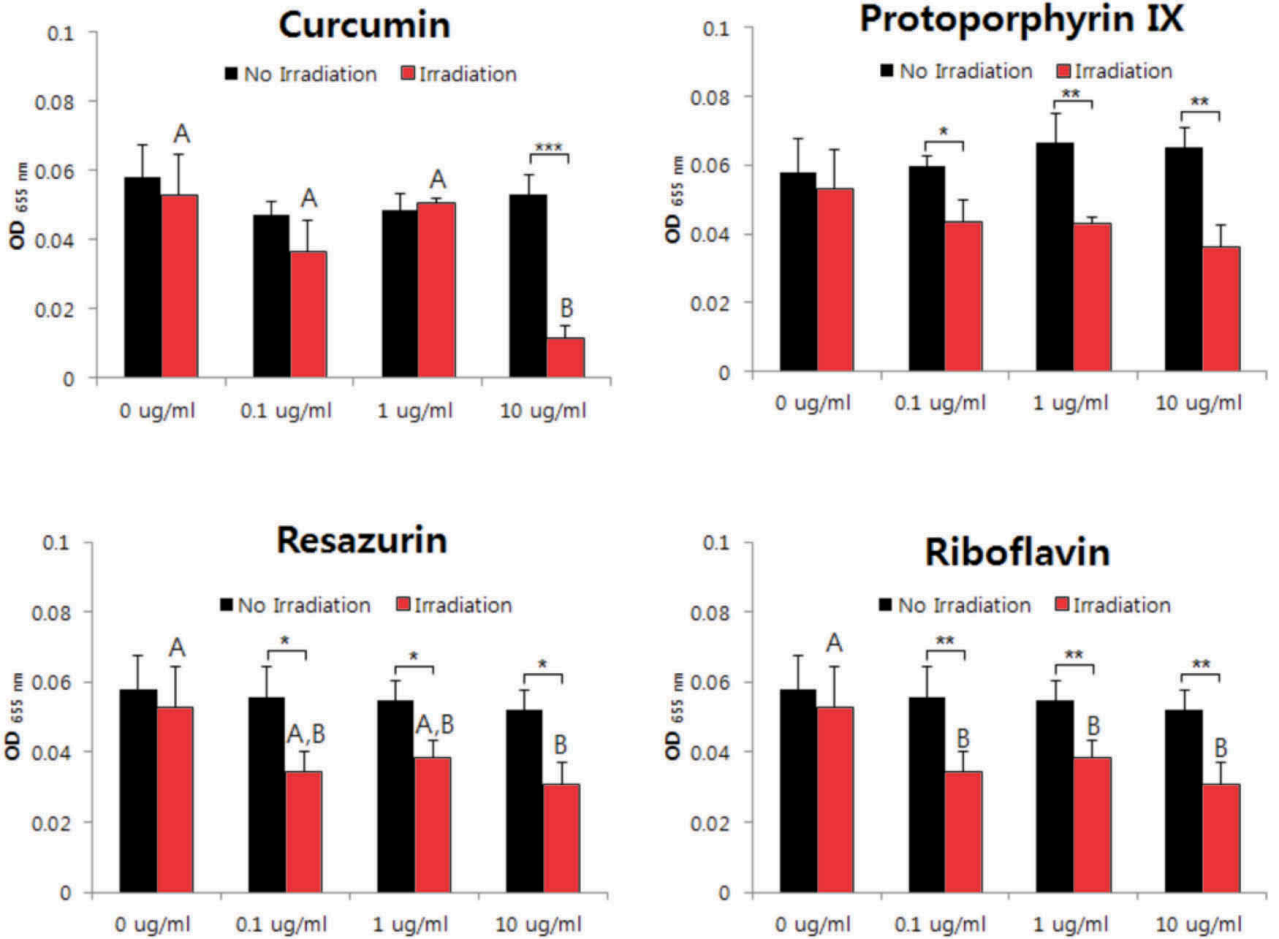
Photodynamic inactivation of *V. parvula* by four PSs. *p < 0.05, **p < 0.01. Different letters indicate significant differences between groups (*p* < 0.05). Data are mean and SD values.

The aPDT effects against *A. israelii* were confirmed for curcumin, protoporphyrin IX, and resazurin. The antibacterial activity was highest for protoporphyrin IX, and the cytotoxicity of the bacteria was identified even for only light irradiation (i.e. without PS) (Figure 1). The aPDT activities against *E. faecium, F. nucleatum*, and *S. mutans* were revealed for only curcumin and protoporphyrin IX. The susceptibility was demonstrated when the protoporphyrin IX concentration was higher than 0.1 μg/ml (Figures 2, 3, and 4). Antimicrobial effects against *L. gasseri* were not observed for any of the four sensitizers at concentrations below 10 μg/ml (Figure 5). The antimicrobial activity against *V. parvula* was confirmed for all four PSs. However, the aPDT activity of curcumin was confirmed only when it was present at a high concentration (10 μg/ml), while the antibacterial activity was evident for the other three PSs all of the concentrations investigated (0.1–10 μg/ml) (Figure 6).

## Discussion

In this study we examined the potential of a short wavelength in the visible light region (405 nm provided by a violet-blue LED) to activate an antibacterial photodynamic reaction with four PSs. Since Akasaki et al. first developed the blue LED [13] it has been used not only for display purposes but also in various medical fields [14–17]. Blue LEDs are used in dentistry for detecting early caries lesion [18] and matured dental plaque based on autofluorescence [19]. The PS currently used for PDT in clinical practice involves irradiation at a wavelength longer than 600 nm [20]. In fact, most PSs are activated by red light, such as hematoporphyrin derivatives (620–650 nm), phenothiazine (620–700 nm), cyanine (600–805 nm), phytotherapeutic agents (550–700 nm), and phytalocyanines (660–700 nm) [21]. This is because PDT involves deep penetration of the skin in order to treat tumors and areas of inflammation [9].

In the field of dentistry there have been several reports on the inhibitory effect of oral microorganisms using PDT. Fonseca et al. reported that a 660-nm diode laser and 0.0125% toluidine blue inhibited the growth of *Enterococcus faecalis* [22], and Metcalf et al. [23] reported that an *S. mutans* biofilm was inhibited by using 500–550 nm light with erythrosine. However, the depth of penetration of light is not a major problem in the oral cavity since most of the relevant microorganisms are present on a tooth surface or the surface of the tongue. In this study we therefore selected PSs that exhibit absorption peaks at 400–500 nm, for curcumin 417 nm, riboflavin 445 nm, and protoporphyrin IX 469 nm, and resazurin 610 nm.

The antimicrobial effect of violet-blue LED and PSs differed with the bacterial species. Among them, *V. parvula* was affected by all PSs in the presence of light irradiation, while *A. israelii* was affected by all PSs except for riboflavin. It was also confirmed that *A. israelii* was inhibited only for light irradiation without PSs, which is probably due to the presence of substances of the porphyrin family in the cells. On the other hand, *E. faecium, F. nucleatum*, and *S. mutans* were found to be suceptible only for curcumin and protoporphyrin IX, and not riboflavin and resazurin. The results for the antimicrobial activities depending on the concentration confirmed that the susceptibility was higher for protoporphyrin IX than curcumin. There was no antimicrobial effect on *L. gasseri* for all four PSs. Generally, aPDT is more effective in inducing inactivation of Gram-positive bacteria, since the outer portion of their cell wall (composed of peptidoglycan and lipoteichoic acid) is relatively more porous, which allows PS to reach the cytoplasmic membrane [24]. However, there was no big difference between Gram-positive and negative bacteria used in this study. Similar to our results, xanthene dyes (such as Rose Bengal and erythrosine) and phenothiazinium dyes (such as toluidine blue O and methylene blue) have also been found to inactivate a wide range of both Gram-positive and Gram-negative bacteria [24,25].

The susceptibility of aPDT also differed according to the types of PSs even for the same bacterial strain. These results have also been found in previous studies. Soria-Lozano et al. reported differences in susceptibility to three sensitizers (methylene blue, rose bengal, and curcumin) for *Streptococcus sanguinis, S. mutans*, and *Candida albicans*, which are caries-related microorganisms [26]. On the other hand, Hakimiha et al. [27] reported no difference in susceptibility to two PSs (toluidine blue O and Radachlorin) against *S. mutans*. This is due to the use of the two PSs at different concentrations. The aPDT efficacy of PSs is governed by the chemical characteristics of each PS, such as hydrophilicity, amphiphilicity, and electric charge. Curcumin and protoporphyrin IX are poorly soluble in water due to their hydrophobic nature. In contrast, resazurin and riboflavin are water soluble due to their hydrophilic nature. Some studies have shown that the hydrophilic cationic photosensitizers show higher effectiveness of aPDT due to their strong attraction to the negatively charged cell membrane and their higher solubility than hydrophobic molecules [28,29]. In addition, hydrophobic molecules are prone to aggregation in physical media, disturbing membrane binding and ROS generation [30,31]. In our previous study, higher concentration (10^4^ ng/mL) of curcumin has been shown to be ineffective in aPDT against *S. mutans* compared to lower concentration [32]. This observation could be due to aggregation of curcumin and its inability to dissolve sufficiently at high concentration. However, our present results showed that hydrophobic PSs are more effective than hydrophilic PSs. Chemically, all PSs are able to absorb light energy and transfer energy [33]. This process relies on the π-conjugation system, which is related to p-orbitals with delocalized electrons in compounds with alternating single and multiple bonds. Among the four PSs which were used in the present study, increasing the length of the π-conjugation also increased the efficiency. These results suggest that different microorganisms in the oral cavity will react differently depending on the type and concentration of specific PSs, the presence or absence of light, and the light wavelength. Further research is therefore needed to selectively control the viability of oral microorganisms.

There are limitations to evaluating planktonic cells of individual strains because many microorganisms in the oral cavity are present within biofilms, and the resistance of a biofilm to antimicrobial agents can be 10- to 100-fold higher than in the planktonic state. Therefore, the effect of these PSs should be evaluated in biofilm models of single strains as well as in multiple-species models including microcosms. Furthermore, many studies that have evaluated the microbial antimicrobial activity through the use of photodynamic reactions (including the present study) still have limitations in inspecting the clinical efficacy. In addition, there has been no comprehensive evaluation of various strains constituting the biofilms present in the oral cavity. Therefore, future validation of PDT for real biofilms should be carried out by utilizing the rapid developments in molecular biology techniques, such as metagenome analysis and metabolomic analysis. In addition, in order to understand how the antibacterial activity differs according to the PS, it is necessary to study the underlying mechanisms in oral microbial cells. Further research is needed for identifying effective treatment methods for the selective control of oral microorganisms while minimizing adverse effects.

## Conclusion

Curcumin, protoporphyrin IX, riboflavin, and resazurin have been found to affect the growth of seven oral microorganisms. However, the effects of different types of PS, concentrations, and light characteristics vary between strains. It is therefore expected that aPDT for selectively inhibiting the growth of oral microorganisms should select the PSs according to the constitution of the oral microbiome in the clinic.
